# Relationship of obesity and insulin resistance with the cerebrovascular reactivity: a case control study

**DOI:** 10.1186/1475-2840-13-2

**Published:** 2014-01-03

**Authors:** Marcela Rodríguez-Flores, Eduardo García-García, Claudia Vanessa Cano-Nigenda, Carlos Cantú-Brito

**Affiliations:** 1 Instituto Nacional de Ciencias Médicas y Nutrición “Salvador Zubirán”, Obesity and Eating Disorders Clinic, SS. Vasco de Quiroga No 15 Col Sección XVI, PC 14000 Mexico City, Mexico; 2 Neurology Department, Instituto Nacional de Ciencias Médicas y Nutrición “Salvador Zubirán”, SS Vasco de Quiroga No 15 Col Sección XVI, PC 14000 Mexico City, Mexico; 3 Internal Medicine Department, Médica Sur Hospital, Mexico City, Mexico

**Keywords:** Cerebrovascular, Insulin resistance, Obesity, Abdominal obesity, Transcranial doppler

## Abstract

**Background:**

Obesity is associated with increased risk for stroke. The breath-holding index (BHI) is a measure of vasomotor reactivity of the brain which can be measured with the transcranial Doppler (TCD). We aim to evaluate obesity as an independent factor for altered cerebrovascular reactivity.

**Methods:**

Cerebrovascular hemodynamics (mean flow velocities MFV, pulsatility index, PI, resistance index, RI, and BHI) was determined in 85 non-obese (Body Mass Index, BMI ≤27 kg/m^2^) and 85 obese subjects (BMI ≥35 kg/m^2^) without diabetes mellitus and hypertension. Anthropometric and metabolic variables, and scores to detect risk for obstructive sleep apnea (OSA) were analyzed for their association with the cerebrovascular reactivity.

**Results:**

The BHI was significantly lower in subjects with obesity according to BMI and in subjects with abdominal obesity, but the PI and RI were not different between groups. There was a linear association between the BMI, the HOMA-IR, the Matsuda index, the waist circumference, and the neck circumference, with the cerebrovascular reactivity. After adjusting for insulin resistance, neck circumference, and abdominal circumference, obesity according to BMI was negatively correlated with the cerebrovascular reactivity.

**Conclusions:**

We found a diminished vasomotor reactivity in individuals with obesity which was not explained by the presence of insulin resistance.

## Background

Obesity is a condition which increases the risk of developing several diseases as a consequence of metabolic derangements and by its mechanical effects [1]. The promotion of insulin resistance and type 2 diabetes confers increased cardiovascular risk in obese subjects [2], which commonly coexist with hypertension and dyslipidemia contributing further to the development of vascular dysfunction and atherothrombotic process [3], Within the atherothrombotic events, cerebrovascular disease is one of the leading diseases for global disease burden [4] and a cause of enormous rates of disability [5]. Data from epidemiological studies has shown hypertension, dyslipidemia, obesity, smoking, and diabetes mellitus to be risk factors for the development of cerebrovascular disease [6].

Among the effects that obesity can have on the cardiovascular system, Obstructive sleep apnea (OSA) is commonly present as weight increases, being reported in over 90% of people with severe obesity [7]; it is associated with sleep fragmentation, somnolence and hypoxemia [8]. Several studies have found an increased risk of developing cerebrovascular disease and myocardial infarction in people with OSA [9,10], as well as an association between persistent hypoxia with inflammation and altered production of adipokines [11].

In order to establish risk of having OSA, the combination of the Epworth somnolence scale (ESS), the sleep apnea clinical scale (SACS) and night oximetry are significantly correlated with polysomnography [12].

It is important to highlight that the reflection of obesity over health can be very variable, according with the studies of obese people without cardiometabolic risk factors, or metabolically healthy [13]. It was reported that body mass index (BMI), abdominal circumference and waist-to-hip ratio did not show to contribute to the prediction of cardiovascular disease better than classical risk factors by a study which calculated hazard ratios from 58 cohorts including individual records of 221,934 people in 17 countries [14]. In contrast, in the assessment of 92 patients (90% of them older than 50 years of age) with ischemic stroke, Singh et al. found BMI to be positively associated with carotid intima-media-thickness [15].

The transcranial Doppler (TCD) allows the non-invasive evaluation of several physiological parameters of the blood flow velocities (BFV) on the main basal intracranial arteries. Its main clinical applications are related with established cerebrovascular disease, malformations, and monitoring during vascular procedures [16]. It provides information on the systolic, diastolic and mean BFV (SBF, DBF, MBF). Measures related with the cerebrovascular resistance are the Goslin’s Pulsatility Index (PI), which is a measure of the variability of BFV in a blood vessel, and the Resistance Index (RI), or Pourcelot Index, which reflects the resistance against the arterial flow originated in the distal microvascular vessels. A retrospective study which performed TCD study in 1208 patients without cerebrovascular disease found that the main factors associated with intracerebral atherosclerosis were age >65 years, smoking, hypertension, and diabetes [17].

The cerebral BFV can also be modified by processes which provoke vasoconstriction or vasodilation, allowing for the evaluation of the cerebrovascular reactivity in response to the injection of acetazolamide, hyperventilation, or breath-holding [18]. Hypercapnia is an important stimulus for cerebral vasodilation, and this condition has allowed the development of a non-invasive test, known as the apnea test, to establish the status of the cerebrovascular reactivity in different clinical conditions [19].

Several studies have utilized the cerebrovascular reactivity to 1) assess the intracranial hemodynamic status in patients with carotid artery disease to predict for the development of cerebrovascular disease; 2) compare the intracranial hemodynamics before and after endarterectomy; 3) compare self-regulation and collateral flow in different regions of the Willis circle; and to 4) predict the development of dementia in patients with cerebrovascular disease [20,21]. One study found a cutoff value of 0.69 to distinguish between normal and abnormal cerebrovascular reactivity in subjects with carotid stenosis [22].

A diminished cerebrovascular reactivity indicates that the reserve self-regulation in the brain is reduced, which has been observed in subjects with diabetes and hypertension [23]. In relation with metabolic syndrome (MetS), Giannopoulos et al. retrospectively analyzed the cerebral vasomotor reactivity in subjects aged 59.19 ± 15. They found an independent association of MetS with reduced cerebrovascular reactivity, even after adjusting for age, gender, race, cardiac disease, current statin therapy, and small vessel disease [24]. Another study evaluated the association of the components of the MetS separately and in combination in 15 volunteers 59 ± 15 years old without evidence of cerebrovascular disease by magnetic resonance. They found a non-significantly increased cerebrovascular reserve calculated after administration of acetazolamide, which was not associated with brain increased oxygen extraction fraction [25].

Taking into consideration the relationship that obesity has with cardiovascular risk factors, it is relevant to know the effect of obesity, in absence of established cardiovascular risk factors (age older than 60 years, hypertension, diabetes, smoking) on the intracranial hemodynamics (PI, RI, and BHI) in comparison with subjects without obesity, to determine if obesity is independently associated with asymptomatic cerebrovascular dysfunction. Secondary objectives are to determine the association of incipient metabolic disorders (insulin resistance, glucose intolerance, dyslipidemia) and high neck circumference with the vasomotor reactivity.

## Research design and methods

We performed an observational, crossectional, case control and prolective study. We included 85 consecutive subjects with obesity Class II and III according to the World Health Organization (WHO) criteria who attended the Obesity and Eating Disorders Clinic at the Instituto Nacional de Ciencias Médicas y Nutrición “Salvador Zubirán” in Mexico City, and 85 subjects without obesity matched by age and gender who signed informed consent. The study was approved by the institution Ethics Committee of the Instituto Nacional de Ciencias Médicas y Nutrición. Inclusion criteria were age 18 to 59 years. Exclusion criteria were recent bariatric surgery (less than two years), weight loss ≥10% of body weight in the last six months, known vascular disease (coronary artery disease, CAD, arrhythmias, congestive heart or kidney failure, cerebrovascular disease, peripheral vascular disease, and carotid stenosis >50%); chronic obstructive pulmonary disease; acute infections; type 2 diabetes mellitus; hypertension; smoking; and inflammatory or autoimmune diseases with vascular effects (arteritis, systemic lupus erithematosus, rheumatoid arthritis).

The study protocol included measurement of weight, height, BMI, abdominal circumference, and neck circumference. Height was measured without shoes using a wall-mounted stadiometer and weight was recorded on a digital scale in a hospital gown. Clinical and metabolic variables included blood pressure, fasting glucose, insulin and lipids, and a 2 hour glucose tolerance test. Glucose intolerance was defined as a 2 hour glucose ≥ 140 mg/dL with a 75 g glucose tolerance test [26]. HOMA-IR and Matsuda indexes were calculated to assess insulin resistance in all subjects [27,28]. Abdominal obesity was determined by the presence of waist circumference >90 cm in men and >80 cm in women, according to the International Diabetes Federation (IDF) [29]. MetS was defined by the presence of three or more of the following: waist circumference >90 cm in men and >80 cm in women, triglycerides ≥ 150 mg/dl; HDL cholesterol < 40 mg/dl in men and < 50 mg/dl in women; systolic blood pressure ≥ 135 mm Hg or diastolic blood pressure ≥ 85 mm Hg; and a fasting glucose ≥ 100 mg/dl, according to the worldwide harmonizing definition of MetS of the International Diabetes Federation (IDF), National Heart, Lung, and Blood Institute (NHLBI), American Heart Association (AHA), World Heart Federation (WHF), International Atherosclerosis Society (IAS), and International Association for the Study of Obesity (IASO) [30]. The Epworth Sleep Scale and the Sleep Apnea Clinical Scale were applied to every subject. Subjects with an ESS score ≥10 or a SACS score ≥15 were considered with risk for OSA.

TCD were performed using a Pioneer TC ultrasound 4040 (Nicolet Biomedical Inc.) Resting velocities in the mean cerebral arteries (MCA) were measured and after ruling out vascular stenosis, cerebrovascular reactivity was determined through the apnea test: During a 30-second apnea, the BFV of the MCA are determined through the temporal or ocular window. Systolic and diastolic values 10 seconds after the apnea phase are registered. For each patient, the pulsatility (PI), resistance (RI) and breath holding indexes (BHI) were calculated, the last for the determination of the cerebrovascular reactivity with the following formulas:

PI = PSV – EDV/MV.

RI = PSV – EDV/PSV.

BHI = 100 × [(_apnea_MV – _resting_MV/(_resting_MV × _apnea_T)] [100] =% cm/seg^19.^

Where: PSV, peak systolic velocity; EDV, end-diastolic velocity; MV, mean velocity (equal to PSV – EDV/3 + EDV); T, time.

### Statistical analysis

The analysis was made with a STATA Data Analysis and Statistical Software Package version 11.1. Sample size was calculated to detect a significant difference in median BHI between the comparison groups, with a power of 90% to detect a difference of 15%, which gives 83 cases in every group. Results are presented as means ± SEM when normally distributed and median (range) when not. Baseline comparisons of continuous and categorical variables in subjects with and without obesity were done by unpaired Student *t* test and chi^2^ test when data were normally distributed, or Mann Whitney U test and Fisher’s exact test for non-parametric, respectively. A simple linear regression analysis was made between the cerebrovascular reactivity and the independent variables (BMI, weight, abdominal circumference, neck circumference, glucose, insulin, insulin resistance indexes, blood lipids, and scores in the ESS and SACS); those with a linear association were included in a stepped multivariable linear regression to adjust for possible confounders associated with the cerebrovascular reactivity. The BHI values were logarithmically transformed before applying a linear regression. A logistic regression was performed to identify factors associated with decreased cerebrovascular reactivity. Decreased BHI was considered as the cut-off value below the 10^th^ percentile of the population without obesity. *P* values <0.05 were considered statistically significant.

## Results

170 subjects were included in the study, 85 with obesity and 85 subjects without obesity. The anthropometric and metabolic variables within each group are shown in Table 1. There was a predominance of women in the sample (80%) given to the increased participation of women in obesity programs. There were significant differences in the metabolic variables between comparison groups, with higher levels of glucose, insulin, and triglycerides, higher values of HOMA-IR, and lower HDL levels and Matsuda Index values in subjects with obesity in comparison with subjects without obesity. There was increased prevalence of metabolic syndrome and risk for OSA according to questionnaires in the obese subjects group as well.

**Table 1:** Characteristics of subjects with and without obesity

	**Non-obese**	**Obese**	**P-value**^**a**^
	**(n = 85)**	**(n = 85)**	
Age, years	36 (26–44)	37 (27–44)	0.97^a^
Female, n (%)	68 (80)	68 (80)	1.00^b^
Anthropometric variables:			
Weight, kg	58.6 (54–62.8)	107.4 (95.6-123.2)	<0.001^a^
Height, m	160 (156–168)	161 (154–167)	0.82^a^
BMI, kg/m^2^	22.9 (21.3-24.7)	41.2 (38.5-45)	<0.001^a^
Waist circumference, cm	76 (71–81.2)	117 (108.5-128)	<0.001^a^
Neck circumference, cm	31 (29–33)	40 (37.8-42)	<0.001^a^
Abdominal obesity, n (%)	15 (17)	84 (100)	<0.001^b^
Metabolic variables:			
Fasting glucose, mg/dL	86 (80–91)	90 (83–97)	0.002^a^
Fasting insulin, mg/dL	7.3 (5.8-9.8)	19 (12.9-26.5)	<0.001^a^
HOMA-IR	1.6 (1.1-2.2)	4.4 (2.9-6)	<0.001^a^
Matsuda index	3.9 (2.2-6.3)	7.8 (5.2-11.1)	<0.001^a^
Glucose intolerance, n (%)	1 (1)	25 (29)	<0.001^b^
Triglycerides, mg/dL	95 (73–131)	138 (102–188.5)	<0.001^a^
Cholesterol, mg/dL	181 (164–203)	173.8 (152.8-201.5)	0.084^a^
HDL, mg/dL	53 (44–62)	42 (35–50)	<0.001^a^
LDL, mg/dL	109 (89.5-122)	101.4 (82.4-121.1)	0.23^a^
Metabolic syndrome, n (%)	2 (2)	33 (38)	<0.001^b^
OSA risk:			
SACS, n (%)	0	4 (4)	0.121^b^
Epworth, n (%)	7 (8)	18 (21)	0.017^b^

Data shown as n (%) or median (Inter Quartile Range). ^a^Mann-Whitney U test; ^b^, *x*^2^, or Fisher exact test as appropriate. BMI, Body Mass Index (weight in kilograms/height in meters squared); HOMA-IR, Homeostasis Model Assessment- Insulin Resistance; LDL, Low Density Lipoprotein Cholesterol; HDL, High Density Lipoprotein Cholesterol; OSA, Obstructive Sleep Apnea; SACS, Sleep Apnea Clinical Scale.

There were no significant differences in the resting mean velocity (MV) in the median cerebral arteries (MCA) in subjects with obesity in comparison to subjects without obesity, respectively. The cerebrovascular reactivity was significantly lower in the group of subjects with obesity in comparison with subjects without obesity. There were no significant differences in the RI or in the PI in subjects without obesity versus subjects without obesity. The same results were found when analyzing according to abdominal obesity (Table 2).

**Table 2:** Doppler velocities and indexes in subjects with and without obesity according to BMI and according to waist circumference

**Artery**	**Velocity/index**	**Non obese (n = 79)**	**Obese (n = 72)**	**P**
**RMCA**	Resting MV, cm/s	59.3 (53.7-66)	60 (52.3-66)	0.73^a^
	PI	0.89 (0.81-1)	0.90 (0.81-1.03)	0.70^a^
	RI	0.56 (0.52-0.6)	0.56 (0.53-0.6)	0.68^a^
	BHI	1.27 (0.94-1.58)	0.83 (0.56-1.21)	<0.001^a^
	Decreased BHI, n (%)	13 (16)	26 (36)	<0.001^b^
		**Non obese (n = 79)**	**Obese (n = 73)**	
**LMCA**	Resting MV, cm/s	59 (55–66.3)	58 (51–66)	0.35^a^
	PI	0.89 (0.8-1)	0.92 (0.79-1)	0.50^a^
	RI	0.56 (0.52-0.6)	0.57 (0.52-0.6)	0.50^a^
	BHI	1.14 (0.92-1.48)	0.89 (0.69-1.11)	<0.001^a^
	Decreased BHI, n (%)	8 (10)	21 (28)	<0.001^b^
		**No abdominal obesity (n = 68)**	**Abdominal obesity (n = 91)**	
**RMCA**	Resting MV, cm/s	60 (53.8-66.3)	60.5 (53–66)	0.58^a^
	PI	0.89 (0.8-1)	0.88 (0.82-1)	0.97^a^
	RI	0.56 (0.52-0.6)	0.56 (0.53-0.6)	0.97^a^
	BHI	1.26 (0.94-1.54)	0.92 (0.64-1.31)	<0.001^a^
	Decreased BHI, n (%)	12 (15)	26 (36)	<0.001^b^
		**No abdominal obesity (n = 64)**	**Abdominal obesity (n = 86)**	
**LMCA**	Resting MV, cm/s	58.8 (54.8-67)	58 (50.7-65.7)	0.58^a^
	PI	0.9 (0.83-0.98)	0.91 (0.78-1)	0.58^a^
	RI	0.56 (0.53-0.6)	0.57 (0.52-0.6)	0.6^a^
	BHI	1.14 (0.93-1.5)	0.94 (0.74-1.18)	<0.001^a^
	Decreased BHI, n (%)	6 (7)	23 (31)	<0.001^b^

Data shown as n (%) or median (Inter Quartile Range). ^a^Mann-Whitney U test; ^b^, *x*^2^, or Fisher exact test as appropriate. RMCA, Right Middle Cerebral Artery; LMCA, Left Mean Cerebral Artery; MV, Mean Velocity; PI, Pulsatility Index; RI, Resistance Index; BHI, Breath Holding Index.

The variables with a significant negative linear association with the cerebrovascular reactivity in a simple linear regression were weight, BMI, waist, neck circumference, and HOMA-IR. The Matsuda Index had a positive linear association with it (Table 3). A multiple linear regression was performed with the cerebrovascular reactivity as the dependent variable and obesity as exposure factor. After adjusting for Matsuda Index, neck circumference, and waist circumference, there was an independent correlation between the cerebrovascular reactivity and obesity according to BMI, with an r^2^ of 0.1 (Table 4). When considering a cut-off value for reduced cerebrovascular reactivity as below the 10^th^ percentile of the BHI in subjects without obesity (0.708), obesity according to BMI was an independent risk factor for diminished cerebrovascular reactivity when adjusting for neck circumference and glucose intolerance (Table 5).

**Table 3:** Simple linear regression of variables associated with the cerebrovascular reactivity

	**r**^**2**^	**β**	**Sβ**	**t**	**p**
Weight	0.06	−0.002	0.001	−3.07	0.003
BMI	0.06	−0.005	0.002	−3.19	0.002
Waist circumference	0.07	−0.002	0.001	−3.39	0.001
Neck circumference	0.05	−0.008	0.003	−2.85	0.005
Fasting glucose	0.02	−0.003	0.002	−1.94	0.05
Fasting insulin	0.03	−0.002	0.001	−2.03	0.04
HOMA-IR	0.03	−0.009	0.005	−1.98	0.05
Matsuda index	0.03	0.007	0.003	2.05	0.04
Glucose intolerance	0.03	−0.093	0.045	−2.08	0.04
Triglycerides	0.02	−0.000	0.000	−1.51	0.13
HDL	0.01	0.001	0.001	1.14	0.26
Somnolence (Epworth)	0.01	−0.062	0.047	−1.3	0.2

**Table 4:** Multiple linear regression of variables associated with the cerebrovascular reactivity

**Variables**	**β**	**Sβ**	**t**	**P**	**CI 95%**	
Obesity	−0.154	0.07	−2.19	0.03	−0.29	−0.015
Matsuda index	−0.002	0.003	−0.74	0.46	−0.007	0.003
Neck circumference	0.001	0.005	0.21	0.83	−0.01	0.012
Waist circumference	0.001	0.001	0.35	0.72	−0.002	0.004

**Table 5:** Logistic regression of variables associated with decreased cerebrovascular reactivity

**Variables**	**OR**	**SE**	**P**	**CI 95%**	
**Obesity**	4.98	3.61	0.03	1.2	20.66
**Neck circumference**	0.97	0.06	0.63	0.86	1.09
**Glucose intolerance**	0.81	0.46	0.72	0.27	2.5

## Discussion

The present study found a significantly reduced cerebrovascular reactivity in young adults with obesity and incipient metabolic disorders, which after adjustment for insulin resistance, glucose intolerance, waist circumference, and neck circumference showed and independent association with elevated BMI.

Several studies have found a poor prediction of cardiovascular outcomes when categorizing people according to BMI [14], so other anthropomethric measures have been evaluated for disease prediction. A significant association between neck circumference with several cardiovascular risk factors has been demonstrated [31], in relation with the respiratory obstruction that an elevated neck circumference produces [32]. The fact that the longitudinal associations between neck and waist circumference with the cerebrovascular reactivity were lost when including BMI and insulin resistance, suggests that although related with hemodynamic affection, an elevated BMI have additional effects on the vasomotor reactivity than the respiratory and metabolic impact that result from the range of neck and waist circumference of the participants.

Inclusion of subjects with moderate and severe obesity confers a risk to include patients with OSA, but excluding patients with hypertension greatly reduces the risk of OSA, since hypertension is one of the most often related conditions [33], and one of the mechanisms by which OSA promotes cardiovascular diseases, in addition to inflammatory status, smooth muscle cell activation, endothelial dysfunction, and increased markers of atherosclerosis [34]. Besides OSA, obesity can affect respiratory physiology in many other ways, including altered work of breathing, ventilatory drive and exercise capacity [35], with the possibility to alter vascular function. Excluding subjects with diabetes also is important for the study of incipient changes in the cerebrovascular function, since it is one of the main conditions that accelerate vascular damage [36]. Nevertheless, in the examination of the cerebrovascular reactivity in subjects with incipient metabolic disturbances, insulin resistance did not show to have an independent association with it. The fact that we found a linear association between different metabolic and anthropometric variables with the cerebrovascular reactivity, but with models reporting low coefficients of correlation for each variable, is consistent with the multiple different mechanisms that can influence the cerebrovascular hemodynamics.

Some studies have explored the development of vascular disorders in association with chronic diseases. One study evaluated the cerebrovascular reactivity in association with cognitive performance and brain volumes in subjects with type 2 diabetes mellitus 56–80 years old at baseline and after four years. Subjects with diabetes had worse cognitive performance and smaller brain volumes in comparison with the control group, but these characteristics did not correlate with the cerebrovascular reactivity, nor with the progressive decline of brain volume in the follow-up [37]. Several reports have shown the association between MetS with ischemic stroke, white matter alteration, and altered brain metabolism in nonelderly adults; and with cognitive dysfunction, in particular in executive function in adolescents [38]. As for obesity alone, one report failed to find statistically significant cognitive impairment associated with obesity [39]. The assessment of the carotid circulation through Doppler ultrasound in subjects free of cardiovascular disease, with and without metabolic syndrome (MS) showed that incidence of cardiovascular events (myocardial infarction, transient ischemic attack, abdominal aortic aneurysm, and thrombo-endo-arterectomy) in a 20-year follow-up was higher among subjects aged 62 ± 12 years old with metabolic syndrome (MetS), and the incidence increased whenever there was presence of preclinical carotid atherosclerosis [40].

The reduction of the cerebrovascular reactivity translates in an altered capacity for vasodilation of the cerebral arteries, which has been associated with the future development of cerebrovascular disease [41]. In this study, the assessment of subjects without established cardiovascular risk factors did not show an advanced disorder in the cerebrovascular hemodynamics, since the PI, which translates distal vascular resistance and is altered in clinical vascular damage [42], was not different in subjects with obesity in comparison with subjects without obesity. The breath holding index is an incipient vascular dysfunction marker, which was negatively related with factors accompanying an elevated BMI. Lack of measurement of all the mechanical and inflammatory mechanisms coexistent with obesity with a vascular repercussion represents a limitation to explain the altered cerebrovascular reactivity that we found.

This study highlights the importance of understanding the implicated mechanisms that an elevated BMI has on the cerebrovascular hemodynamics in young adults, in absence of other established risk factors for stroke. Additional studies are needed to discover the different processes that determine a clinical event in the presence of this alteration of the cerebrovascular reactivity, and to examine the interactions of multiple phenomena contributing with the development of cerebrovascular disease.

## Competing interests

None of the authors has any competing interest to declare.

## Authors’ information

CCB is Professor of the high specialty course on Cerebrovascular Disease for Neurologists at the Instituto Nacional de Ciencias Médicas y Nutrición.

MRF is currently Chair of the Latin American Affairs Section of the Obesity Society.

## Authors’ contributions

CCB, MRF and EGG worked on the study design, VCN and MRF carried out the clinical evaluations, MRF and CCB analysed the data. MRF wrote the article. All authors read and approved the final manuscript.
